# The Small RNA-Binding Protein CcaF1 Promotes Formation of Photosynthetic Complexes in *Rhodobacter sphaeroides*

**DOI:** 10.3390/ijms24119515

**Published:** 2023-05-30

**Authors:** Julian Grützner, Janek Börner, Andreas Jäger, Gabriele Klug

**Affiliations:** Institute of Microbiology and Molecular Biology, Justus Liebig University Giessen, Heinrich-Buff-Ring 26-32, 35392 Giessen, Germanyjanek.boerner@mikro.bio.uni-giessen.de (J.B.); andreas.jaeger@mikro.bio.uni-giessen.de (A.J.)

**Keywords:** RNA-binding proteins, riboregulation, *Rhodobacter*, RNA stability, phototrophic growth, RIPseq

## Abstract

In natural habitats, bacteria frequently need to adapt to changing environmental conditions. Regulation of transcription plays an important role in this process. However, riboregulation also contributes substantially to adaptation. Riboregulation often acts at the level of mRNA stability, which is determined by sRNAs, RNases, and RNA-binding proteins. We previously identified the small RNA-binding protein CcaF1, which is involved in sRNA maturation and RNA turnover in *Rhodobacter sphaeroides. Rhodobacter* is a facultative phototroph that can perform aerobic and anaerobic respiration, fermentation, and anoxygenic photosynthesis. Oxygen concentration and light conditions decide the pathway for ATP production. Here, we show that CcaF1 promotes the formation of photosynthetic complexes by increasing levels of mRNAs for pigment synthesis and for some pigment-binding proteins. Levels of mRNAs for transcriptional regulators of photosynthesis genes are not affected by CcaF1. RIP-Seq analysis compares the binding of CcaF1 to RNAs during microaerobic and photosynthetic growth. The stability of the *pufBA* mRNA for proteins of the light-harvesting I complex is increased by CcaF1 during phototrophic growth but decreased during microaerobic growth. This research underlines the importance of RNA-binding proteins in adaptation to different environments and demonstrates that an RNA-binding protein can differentially affect its binding partners in dependence upon growth conditions.

## 1. Introduction

Members of the genus *Rhodobacter* are characterized by high metabolic versatility, which allows them to adapt to changing environmental conditions. While aerobic respiration takes place in the presence of oxygen, anoxygenic photosynthesis (in the presence of light), anaerobic respiration, or fermentation (both in the absence of light) can generate ATP under anoxic conditions. Many studies have addressed the adaptation of *Rhodobacter capsulatus* and *Rhodobacter sphaeroides* (now renamed *Cereibacter* [1]) to changing oxygen and light conditions in the past (reviewed in [2,3,4]). Several proteins directly or indirectly affecting the rates of transcription of photosynthesis genes in response to oxygen levels were identified (the names of the *R. capsulatus* proteins given in parentheses): the two-component system PrrA/PrrB (RegA/RegB) [5,6], the repressor–anti-repressor systems PpsR (CrtJ)/AppA and PpaA (AerR) [7,8,9], and FnrL [10,11]. AppA and PpaA (AerR) do not only function as oxygen-sensors but also as photoreceptors that sense light via a heme or cobalamin cofactor [8,12,13,14,15].

Besides this protein-based regulation, riboregulation has an important role in the control of photosynthesis gene expression in *Rhodobacter*. More than two decades ago, the differential stabilities of segments of the polycistronic *puf* operon (encoding components of the photosynthetic complexes) of *R. capsulatus* were reported to affect the ratios of the reaction center (RC) and light-harvesting (LH) I complexes [16]. Later on, the important roles of the *R. sphaeroides* sRNAs PcrZ, PcrX, and asPcrL in regulated photosynthesis gene expression were demonstrated [17,18,19]. Hfq, an RNA chaperone interacting with many sRNAs, contributes to the photo-oxidative stress resistance of *R. sphaeroides* and also affects its levels of photosynthetic complexes [20,21]. In addition, a remarkable strong effect of the endoribonuclease RNase E on phototrophic but not on chemotrophic growth was observed [22]. RNase E was shown to differentially affect the stability of mRNAs for the important regulators AppA and PrrB under microaerobic and phototrophic growth conditions [23].

**Figure 1:**
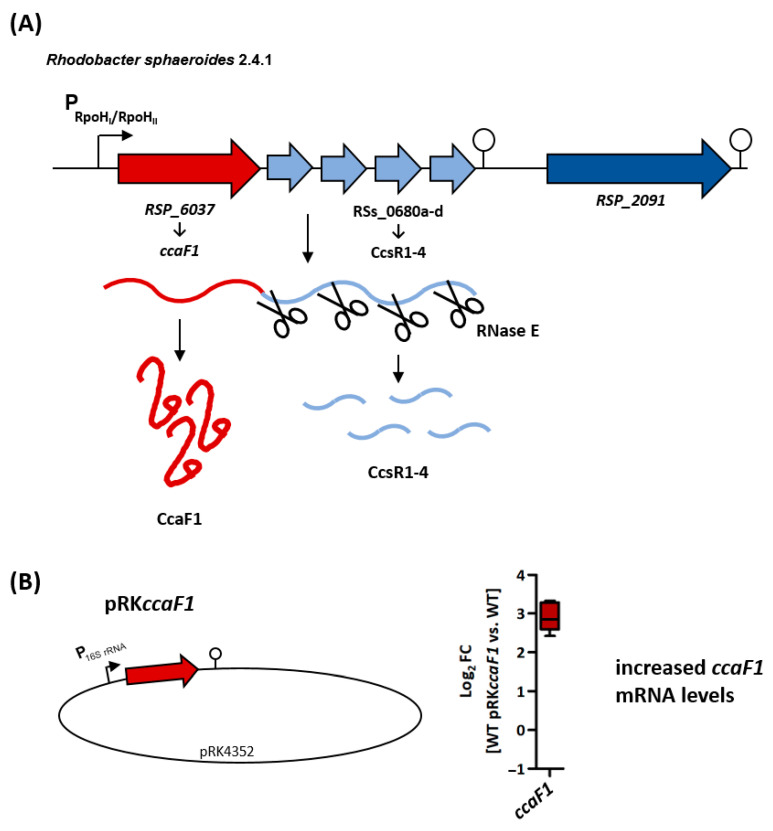
(**A**) Genomic context of the DUF1127 protein CcaF1 (dark red) and the CcsR1-4 sRNAs (light blue) from *R. sphaeroides* 2.4.1. The protein–sRNA operon is preceded by an RpoH_I_/RpoH_II_ promoter (black arrow), and a Rho-independent terminator structure is located at the 3′ end (modified from [25]). The transcribed RNA precursor is processed by the endoribonuclease RNase E into the *ccaF1* mRNA and the CcsR1-4 sRNAs [24]. (**B**) Schematic overview of the plasmids introduced in the *R. sphaeroides* wild type. In strain WT pRK*ccaF1*, the *ccaF1* gene is transcribed from a strong 16S rRNA promoter, leading to a strongly increased *ccaF1* mRNA level. This finding was confirmed by a quantitative real-time RT-PCR with total RNA samples from biological triplicates of the *ccaF1* overexpression strain (WT pRK*ccaF1*) and the empty vector control, as shown in the right panel.

Recently, we identified a new type of small RNA-binding protein in *R. sphaeroides*: CcaF1 (conserved CcsR associated factor 1) [24]. The *ccaF1* gene is co-transcribed with four CcsR RNAs that modulate the C1 metabolism and have an important role in stress defense [25] (Figure 1A). *ccaF1* encodes a small protein of 71 amino acids that comprises a DUF1127 domain. An association of short DUF1127 proteins with CcsR-like sRNAs is often found in alphaproteobacteria and was labeled CIN1 locus [26]. The CcaF1 protein of *R. sphaeroides* affects CcsR levels in trans and alters stress resistance. Together with RNase E, CcaF1 is involved in the processing of the *ccaF1*–CcsR transcript and in the maturation of the CcsR RNAs. But CcaF1 also affects levels of many other RNAs in *R. sphaeroides*, including RNAs for photosynthesis genes, and it affects the stability of the *pufBA* mRNA. Overexpression of *ccaF1* strongly impedes growth under microaerobic conditions [24]. 

Based on these findings, we were interested in the role of CcaF1 in phototrophic growth. Our data demonstrate that CcaF1 has a mild promoting effect on doubling time during phototrophic growth and influences the transition of *R. sphaeroides* from chemotrophic to phototrophic growth. Co-immunoprecipitation demonstrates that CcaF1 directly binds to several mRNAs for proteins with a direct function in photosynthesis.

## 2. Results

### 2.1. CcaF1 Affects Phototrophic Growth and Pigmentation of R. sphaeroides

It was not possible to generate a viable strain lacking *ccaF1* [25]. Therefore, we compared strain WT pRK*ccaF1* (wild type carrying the *ccaF1* overexpression plasmid) to strain WT pRK4352 (empty vector control) [24]. Plasmid pRK4352 carries a strong rRNA promoter from *R. sphaeroides;* in plasmid pRK*ccaF1*, the *ccaF1* gene is transcribed from this promoter, leading to strongly increased *ccaF1* mRNA levels (Figure 1B). Strain WT pRK*ccaF1* was strongly impeded in growth compared to the wild type or control strain WT pRK4352 when incubated under microaerobic conditions [24].

**Figure 2:**
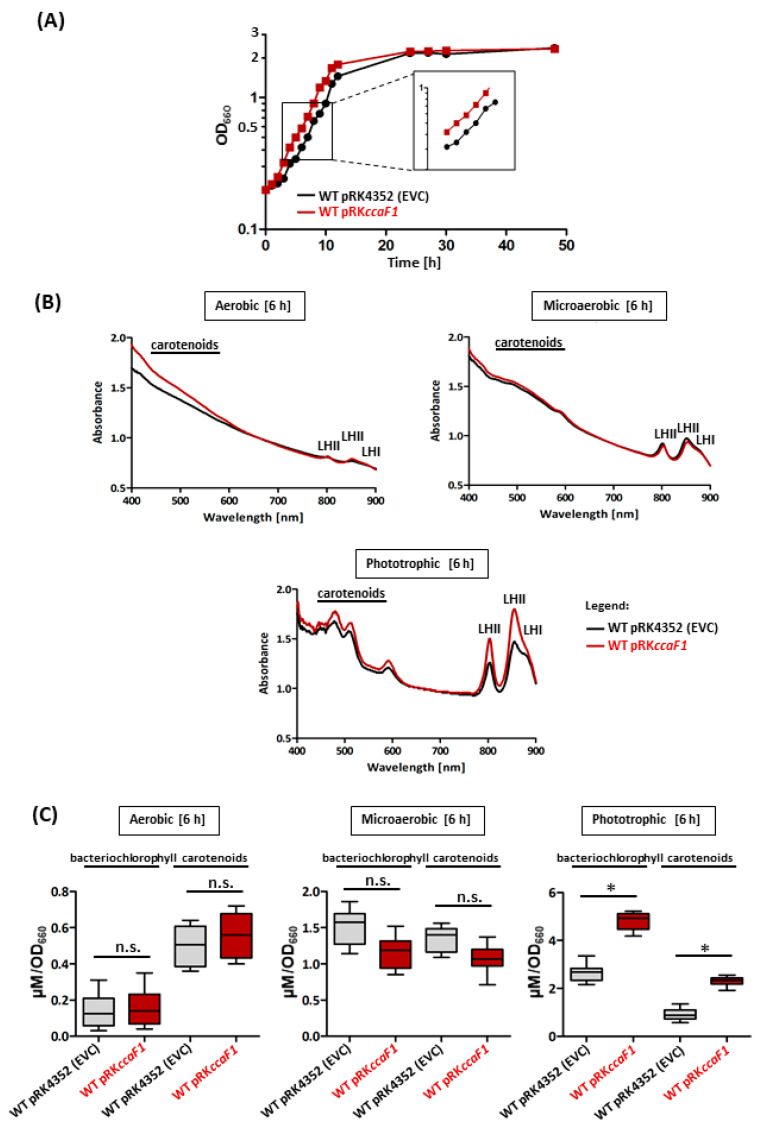
(**A**) Growth behavior of *R. sphaeroides* WT pRK4352 (EVC; black) or *R. sphaeroides* with the *ccaF1* overexpression plasmid (pRK*ccaF1*; red) was monitored over 48 h. The plotted optical densities at 660 nm (OD_660_) represent the mean of at least three independent experiments. The standard deviation of the mean was calculated (error bars are not visible due to a high reproducibility). (**B**) Whole-cell spectra were analyzed for the empty vector control and the *ccaF1* overexpression strain (pRK*ccaF1*; red). Microaerobic overnight cultures were diluted on the next day to an OD_660_ 0.2 and incubated further under aerobic, microaerobic, or phototrophic growth conditions for 8 h (See bacterial strains and growth conditions). After 8 h under aerobic, microaerobic, or phototrophic growth conditions, the absorbance was measured from 400 nm to 900 nm. The peaks at 800 and 850 nm represent specific absorbance maxima of the light-harvesting complex II (LHII), and the peak at 870 nm stems from absorbance of the light-harvesting complex I (LHI). The absorbance of the reaction center at 800 and 870 nm is covered by the absorbances of the more abundant LH complexes. Shown are results of three independent experiments, each performed in technical duplicates. (**C**) The bacteriochlorophyll and carotenoid contents in the *R. sphaeroides* WT *ccaF1* overexpression strain (pRK*ccaF1*; red) or the empty vector control (pRK4352; gray) were analyzed. To calculate the bacteriochlorophyll content (µM), the absorbance was normalized to the OD_660_, and the extinction coefficient of 76 mM^−1^ cm^−1^ was applied. The carotenoid-specific absorbance was measured at 484 nm. To calculate the carotenoid content (µM), the absorbance was normalized to the OD_660_, and the extinction coefficient of 128 mM^−1^ cm^−1^ was applied. Shown are results of nine independent experiments, each performed in technical duplicates (mean values and their standard deviation are shown). Student’s two-sided t-test was applied to assess the statistical significance of differences in pigment amount mean values between EVC and *R. sphaeroides* pRK*ccaF1* (*: *p* < 0.05; n.s.: not significant).

When the strains were cultivated microaerobically and then shifted to phototrophic growth, doubling of WT pRK*ccaF1* was slightly faster in the exponential phase than the control (Figure 2A). Both strains reached a similar optical density in the stationary phase.

Figure 2B shows that after 6 h under phototrophic growth, the strain overexpressing *ccaF1* accumulated higher amounts of photosynthetic complexes than the EVC. After 6 h under microaerobic conditions, in which the formation of photosynthetic complexes in *Rhodobacter* is already induced, the absorption spectra were almost identical between the overexpressing strain and the control (Figure 2B). 

A quantitative analysis of the bacteriochlorophyll and carotenoid levels in both strains under the different growth conditions is shown in Figure 2C. While the differences in bacteriochlorophyll and carotenoid content between the two strains were not significantly different under aerobic and microaerobic conditions, significantly higher pigment levels were observed under phototrophic conditions in the pRK*ccaF1*-carrying strain (bacteriochlorophyll levels increased by a factor of 2.1, carotenoid levels by a factor of 2.8) compared to the EVC.

### 2.2. Levels of Some mRNAs for Pigment Synthesis and for Pigment-Binding Proteins Are Affected by CcaF1

**Figure 3:**
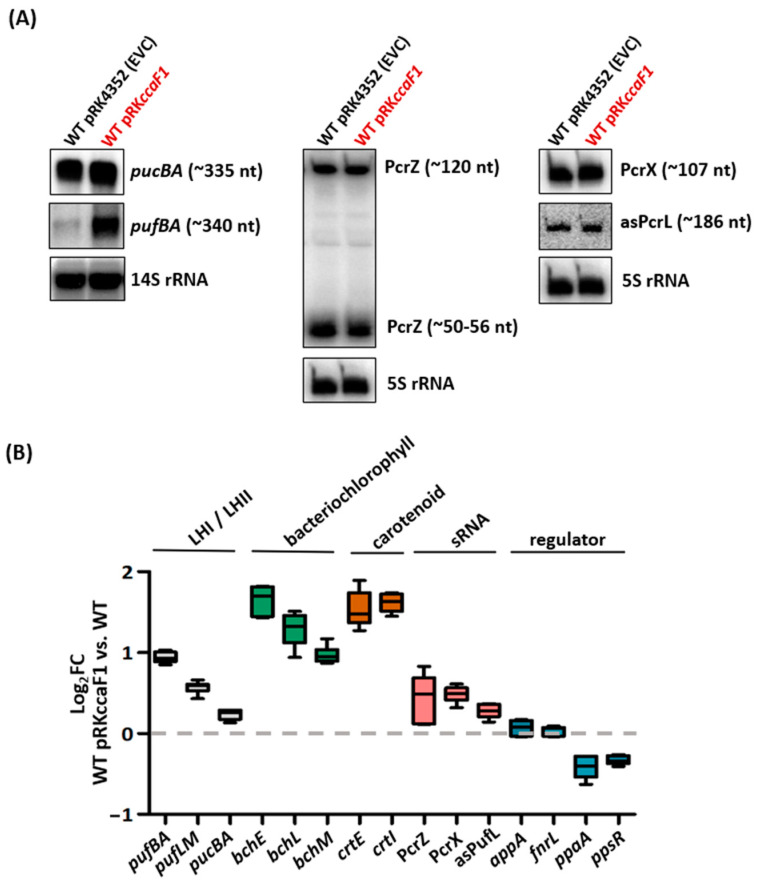
(**A**) Northern blot analysis of *R. sphaeroides* WT pRK4352 (EVC) and *ccaF1* overexpression (pRK*ccaF1*). Samples were taken after 8 h under phototrophic growth conditions, and total RNA was isolated. For detection of mRNA transcripts (*pufBA* and *pucBA*), 10 µg total RNA were separated on a 1% agarose (*w/v*) formaldehyde gel. The 14S rRNA served as a loading control. *R. sphaeroides* cleaves the 23S RNA into fragments of 16S and 14S. For the detection of small RNAs (PcrZ, PcrX, asPufL), 8 µg total RNA were separated on a 10% polyacrylamide gel containing 7 M urea. The 5S rRNA served as a loading control. (**B**) The abundance changes of mRNAs encoding pigment-binding proteins of the LHI/II complex (*pufBA*, *pucBA*), bacteriochlorophyll synthesis (*bchE*, *bchL*, *bchM*), carotenoid synthesis (*crtE*, *crtI*) and photosynthesis regulators (*appA*, *fnrL*, *ppaA*, *ppsR*), and the sRNA PcrZ were analyzed by quantitative real-time RT-PCR. In addition, 20 ng DNA-free total RNA were used for real-time RT-PCR. Changes in abundance were normalized to *rpoZ* (housekeeping gene). Shown are results of three independent experiments, each performed in technical duplicates.

To form photosynthetic complexes, the pigment-binding PufBALM and PucBA proteins and the assembly factor PufX, as well as photopigments, are required. Previous analyses under microaerobic conditions demonstrated an effect of CcaF1 on the level and stability of the *pufBA* mRNA [24]. Figure 3A shows northern blots for total RNA from cells isolated after 6 h of phototrophic growth and hybridized either against a *pufBA* or *pucBA* DNA probe. In strain WT pRK*ccaF1,* the levels of the *pufBA* mRNA-encoding pigment-binding proteins of the LHI complex were clearly increased in comparison to the control strain. Such an increase was not observed for the *pucBA* mRNA, encoding proteins of the LHII complex. We also analyzed the effect of CcaF1 on the abundance of sRNAs with a known function in the regulation of photosynthesis gene expression. Northern blots revealed a minor change in PcrZ levels, which is processed to a stable smaller 3′ fragment [17]; in PcrX that is processed from the 3′ UTR of the *pufBALMX* mRNA [18]; and in asPufL, which is antisense to a part of *pufL* [19]. These observations were also confirmed by real time RT-PCR (Figure 3B), which revealed increased levels of mRNAs for bacteriochlorophyll synthesis: *bchE* (encoding magnesium-protoporphyrin IX monomethyl ester oxidative cyclase), *bchL* (light-independent protochlorophyllide reductase), and *bchM* (Mg-protoporphyrin IX methyl transferase), which belongs to two different operons. Likewise, increased mRNA levels upon overexpression of *CcaF1* were shown for two genes for carotenoid synthesis: *crtE* (geranylgeranyl pyrophosphate synthetase) and *crtI* (phytoene dehydrogenase), belonging to different operons. The *pufLM* mRNA encoding the L and M proteins of the photosynthetic reaction center showed a slight increase in the overexpression strain, as well as the sRNA PcrX that is co-transcribed with the *pufBALM* genes. Real-time RT-PCR also revealed slightly increased levels of PcrZ and asPufL upon overexpression of *ccaF1*; slightly decreased levels of *ppaA* and *ppsR*; and no effect on levels of *appA* and *fnrL* (Figure 3B). This finding suggests that the increased amounts of photosynthetic complexes in the overexpression strain is rather due to increased mRNA levels for Puf proteins and for pigment synthesis than to altered mRNA levels for transcription regulators of photosynthesis genes. 

### 2.3. CcaF1 Increases the Half-Life of pufBA under Phototrophic Growth

**Figure 4:**
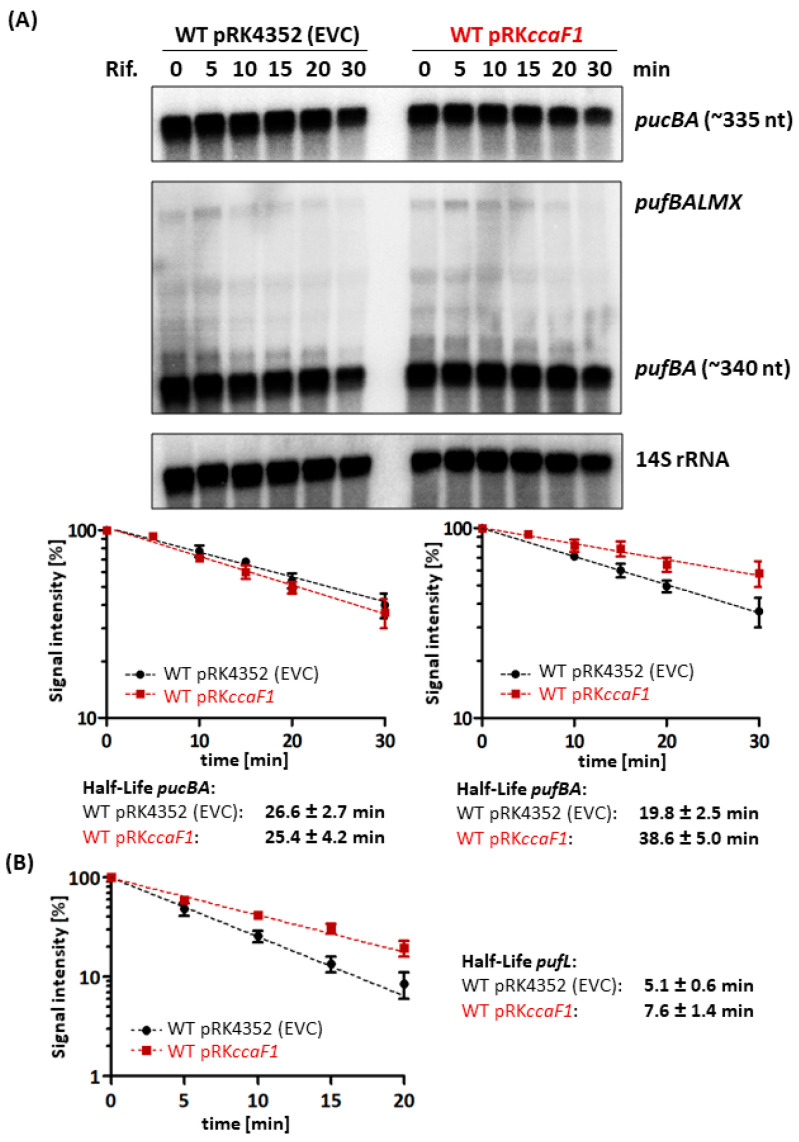
Determination of *pucBA*, *pufBA*, and *pufL* mRNA half-lives in the empty vector control (pRK4352) and upon *ccaF1* overexpression (pRK*ccaF1*). Samples were taken at different time points after adding rifampicin (Rif.). The average half-life was calculated from three independent experiments, and the standard deviation is indicated. (**A**) Total RNA was isolated and separated on a 1% agarose formaldehyde gel. After blotting, the mRNAs of *pucBA*, *pufBALMX*, and *pufBA* were hybridized to specific radio-labelled PCR-products. For quantification, RNA signal intensities were normalized to signals of the 14S rRNA loading control. (**B**) To quantify *pufL*-specific mRNA levels, 20 ng DNA-free total RNA and *pufL*-specific primers were used for real-time RT-PCR. Changes in abundance were normalized to *rpoZ* (housekeeping gene).

Our previous study revealed that overexpression of *ccaF1* under microaerobic conditions decreased the *pufBA* mRNA half-life from about 22 min to only 12 min, while there was no significant effect on the *pucBA* mRNA (18–20 min half-life) [24]. Figure 4A shows northern blots for phototrophic cultures. While the half-life of *pucBA* was about 26 min for both strains, the half-life of the *pufBA* mRNA was almost doubled in WT pRK*ccaF1* (about 39 min versus about 20 min in the control) (Figure 4A). Under microaerobic conditions, overexpression of *ccaF1* resulted in decreased *pufBA* mRNA half-life [24]. 

As seen in Figure 4A, the *pufBALMX* mRNA is hardly detectable by northern blot. Therefore, we monitored the decay of the *pufL* segment by using real-time RT-PCR (Figure 4B). While the half-life of the shorter *pufBA* fragment was strongly stabilized upon overexpression of *ccaF1*, only a moderate stabilizing effect was observed for the *pufL* segment (8 min versus 5 min in the control). 

### 2.4. CcaF1 Interacts with Some Photosynthesis mRNAs, but Not with mRNAs for Transcriptional Regulators

**Figure 5:**
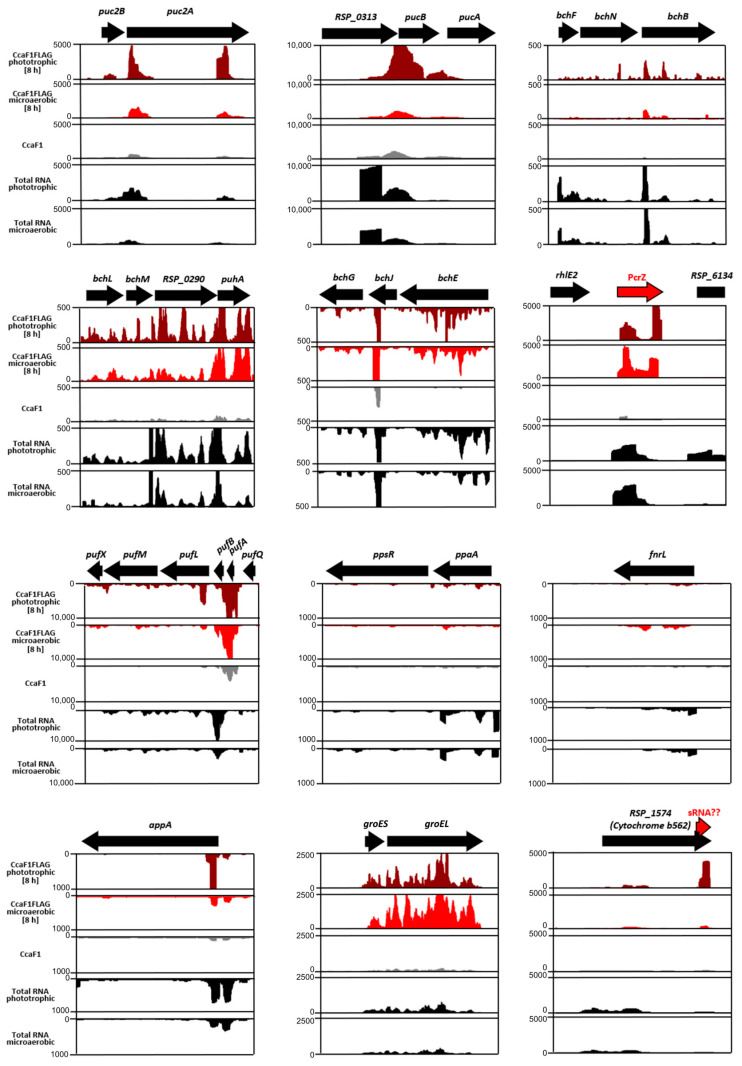
Analysis of co-immunoprecipitated RNA using CcaF1 with 3xFLAG-tag (CcaF1FLAG) or without 3xFLAG-tag (CcaF1, untagged control) on the plasmid pRK4352 by RNA-Seq (RIP-Seq). The CoIP was performed as described in [24]. Read coverage plots from the Integrated Genome Browser display the sequencing reads for selected RNAs. The read coverage plots of CcaF1FLAG under phototrophic growth conditions are shown in dark red, under microaerobic growth conditions in red, CcaF1 without FLAG-tag (control) in grey, and total RNA under microaerobic and phototrophic growth conditions in black.

We applied co-immunoprecipitation (CoIP) to test for a direct interaction of CcaF1 and mRNAs for pigment-binding proteins, enzymes of pigment synthesis, or regulatory proteins and sRNAs under phototrophic conditions. After sequencing of samples obtained from CoIP (RIP-Seq), the data were processed into wiggle files and visualized in the Integrated Genome Browser (screenshots displayed in Figure 5). We compared this data set to the RIP-Seq results under microaerobic conditions and to a CoIP with non-tagged CcaF1 (negative control). At the bottom, RNA-seq data from total RNA, both under microaerobic and phototrophic conditions is shown. 

Six hours after transition to phototrophic growth, we observed higher levels of *pucBA, puc2BA, bchN, bchB, bchL, bchM* mRNAs (from the same operon), *bchE, bchJ,* and *bchG* mRNAs (from the same operon) in CoIP RNA, compared to RIP-Seq from microaerobic cultures. *R. sphaeroides* harbours two *pucBA* operons [27]. The Puc2A protein is much longer than PucA and is not assembled into LHII complexes like PucA, PucB, and Puc2B [28]. Moreover, *pufBA* showed similar enrichment in phototrophic and microaerobic conditions, but the read coverage pattern was somewhat different.

The 3′ segment of sRNA PcrZ was more enriched in the RIP-Seq under phototrophic than under microaerobic conditions. A remarkable strong enrichment under phototrophic conditions was observed for the 3′ part of *RSP_1574* (mRNA for cytochrome b562 from the cytochrome bc1 complex that is involved in respiratory and cyclic photosynthetic electron transport). The *groEL* mRNA is shown as an example for an mRNA that is more enriched under microaerobic conditions. The *ccaF1* mRNA level was increased in total RNA from phototrophic cultures compared to chemotrophic cultures (Figure S1).

Figure S2 shows gels of the CoIP RNAs and confirms the interaction of CcaF1 to *pufBA, pucBA, bchB, bchE, bchN, bchM*, and PcrZ. The mRNAs for the regulators of photosynthesis genes FnrL, PpaA, and PpsR showed no interaction with CcaF1. There was a strong enrichment for the 3′ end of the *appA*-coding mRNA but not for the downstream region.

**Figure 6:**
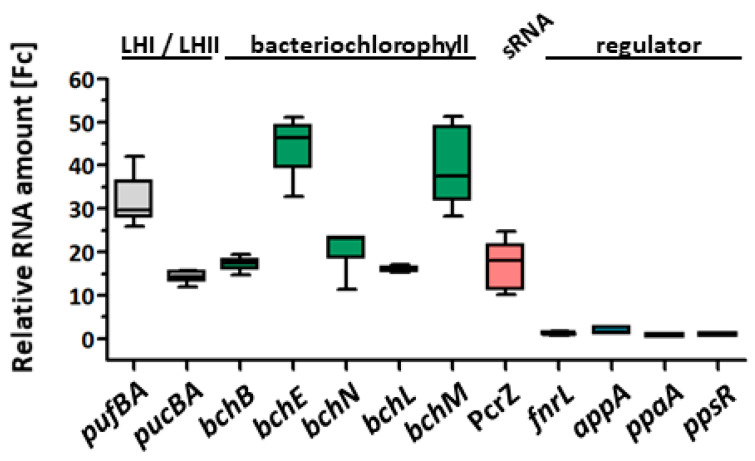
Validation of co-immunoprecipitated RNA by real-time RT-PCR using CcaF1 with 3xFLAG-tag (CcaF1FLAG) or without 3xFLAG-tag (CcaF1, untagged control) on the plasmid pRK4352. 20 ng DNA-free total microaerobic overnight cultures were diluted on the next day to an OD_660_ 0.2 and incubated under phototrophic growth conditions for 8 h (see bacterial strains and growth conditions). The RNA from independent biological triplicates was analyzed. The fold change between flag-tagged CcaF1 versus the non-flag-tagged CcaF1 (control) samples is plotted.

We quantified the enrichment of RNAs in the CoIP samples with real-time RT-PCR (Figure 6). This approach confirmed high enrichment factors (14–43 fold) for *pufBA*, *pucBA*, *bch* mRNAs, and the sRNA PcrZ in the CoIP. While mRNAs for the regulators FnrL, PpaA, and PpsR were not enriched, the 5′ part of *appA* showed a 1.8-fold enrichment.

## 3. Discussion

Bacteria have a remarkable ability to cope with different environmental conditions by adjusting their metabolism and by mounting stress responses. This requires regulation of gene expression and involves a multitude of different mechanisms to adjust the transcriptome and the proteome. These mechanisms of regulation also include RNA-binding proteins that often work together with sRNAs (e.g., [29]). The role of some bacterial RNA-binding proteins has been intensely studied in the past. The small Hfq protein (77 amino acids, as in *R. sphaeroides*) is a global regulator of sRNA-based networks in many bacterial species, and deletion mutants of Hfq have often pleiotropic phenotypes [30]. Such pleiotropic effects are also described for *Rhodobacter*, including an influence on the formation of pigment-protein complexes [20]. The FinO domain protein ProQ was later identified as another global RNA-binding protein that interacts with many sRNAs and mRNAs [31]. So far, no ProQ homolog was identified in *R. sphaeroides*. Recently, KH-domain proteins were also shown to be important for sRNA function [32]. Small proteins with DUF 1127 domains have been found in many bacterial species, but their interaction with RNAs was so far only reported for *R. sphaeroides* [24].

This study demonstrates a differential impact of CcaF1 on gene expression and growth during chemotrophic or phototrophic cultivation. While overexpression of *ccaF1* strongly impedes chemotrophic growth under microaerobic conditions [24], it has a small growth-promoting effect on phototrophic cultures. Thus, CcaF1 contributes to the transition of *R. sphaeroides* between different growth conditions. 

The slight growth-promoting effect of CcaF1 under phototrophic conditions is linked with increased amounts of photosynthetic complexes and increased levels of mRNAs that are required for pigment synthesis or synthesis of the *puf* mRNA for the reaction center and LHI proteins. Surprisingly, an increase of the *pucBA* mRNAs for LHII proteins was not observed. LHII is the most abundant complex under photosynthetic conditions [33]. The ratio of RC and LHI complexes is relatively constant (about 1:14). Interestingly, this ratio is a consequence of the differential stabilities of segments of the polycistronic *pufBALMX* mRNA. Deletion of a hairpin loop that stabilizes the 3′ end of the *pufBA* mRNA results in altered ratios of RC to LHI [16]. The ratio of LHII to the RC–LHI core complexes is flexible and also influenced by growth conditions [34]. After a shift from chemotrophic to phototrophic growth, *puf* and *puc* mRNAs are induced; RC–LHI complexes occur first, and then LHII associates to the RC–LHI core complexes [35]. For the increased formation of LHII complexes upon overexpression of *ccaF1*, as seen in the spectra in Figure 2B, obviously no overproduction of the *pucBA* mRNA is required. It is conceivable that the *pucBA* levels produced without *ccaF1* overexpression are sufficient for this overall increase, which is controlled by RC–LHI complexes into the membrane.

The increased levels of *pufBA* mRNA in the presence of higher amounts of CcaF1 are linked with an increased half-life under phototrophic conditions. CcaF1 was shown to promote RNase E-mediated maturation of the CcsR1 RNAs but also to decrease the half-life of several tested RNAs (including that of *pufBA*) under microaerobic conditions. The half-lives of other RNAs that were shown to bind to CcaF1 by CoIP were not affected [24], as also shown in Figure 4 for *pucBA* mRNA under phototrophic conditions. This implies that CcaF1 can promote degradation of bound RNA but can also stabilize RNA or not affect the half-life. This effect depends most likely on the features of the RNA targets, like the presence and localization of RNase E cleavage sites. It is surprising that the effect of CcaF1 on *pufBA* stability is the opposite in microaerobic and phototrophic conditions. It is unlikely that the target RNAs adopt significantly different structures under the different growth conditions. It is known that binding to the membrane has a strong influence on RNase E activity in the gammaproteobacterium *E. coli*, while in the alphaproteobacterium *Caulobacter crescentus* RNase E localizes to BR bodies [36]. Data on the localization of RNase E in the alphaproteobacterium *Rhodobacter* are not available, as the *Rhodobacter* enzyme possesses no membrane-targeting sequence. Under microaerobic as well as under phototrophic conditions, photosynthetic complexes are formed and inserted into intracytoplasmic membrane vesicles that are absent in aerobic cultures. As seen in Figure 2B, the amounts of photosynthetic complexes that correlate to the amounts of intracytoplasmic membranes are higher under phototrophic growth. It is, however, unlikely that this difference would cause opposite effects of CcaF1 on the stability of certain targets. It is likely that further yet unknown factors are involved in the CcaF1–target RNA–RNase E interplay that still need identification. 

Remarkably, the amounts of mRNAs for known regulators of the formation of components of the photosynthetic apparatus (AppA, FnrL, PpaA, PpsR) and for PcrX were not or were only slightly affected by CcaF1. This underlines that CcaF1 directly affects the amounts of some of its binding partners by affecting stability and that the changes in the amounts of *pufBA*, *bch*, and *crt* mRNAs are not the consequence of an indirect effect by these regulators. This finding emphasizes the important role of RNA-binding proteins in the regulation of bacterial gene expression and in bacterial adaptation.

## 4. Materials and Methods

### 4.1. Bacterial Strains and Growth Conditions

All *R. sphaeroides* strains are listed in Table S1 and were cultivated in malate minimal-salt medium or on solid medium containing 1.6% (*w*/*v*) agar at 32 °C in the dark [37]. For strain construction see Grützner et al. [24]. When necessary, tetracycline (2 µg mL^−1^) was added to liquid and solid growth media. 

For all experiments (growth behavior, pigment analysis, and determination of RNA half-life) Erlenmeyer flasks were filled up to 80% with malate minimal-salt medium, inoculated 1% with the corresponding *R. sphaeroides* strain (see Table S1), and incubated overnight under microaerobic growth conditions (140 rpm, 32 °C). The microaerobic preculture with an OD_660_ around 0.6 was diluted on the next day to an OD_660_ 0.2 and shifted to aerobic (baffled Erlenmeyer flasks were filled up to 25% and shaken at 180 rpm, 32 °C), microaerobic (Erlenmeyer flasks were filled up to 80% and shaken at 180 rpm, 32 °C), or phototrophic (sealed Meplat bottles were filled up to 100% and illuminated with 60 W/m^2^ white light at 32 °C) growth conditions. 

To monitor the growth behavior, the optical density (OD) was measured every 1.5 h at 660 nm (Specord 50, Analytic Jena AG, Jena, Germany).

### 4.2. Pigment Analysis

For pigment analysis, *R. sphaeroides* strains were cultivated overnight under microaerobic growth conditions, diluted to an OD_660_ 0.2, and incubated for 8 h under aerobic, microaerobic, or phototrophic growth conditions (see bacterial strains and growth conditions). For whole-cell absorbance spectra, 1 mL of the culture was transferred into a cuvette, and the absorbance was measured in a spectral photometer at wavelengths of 400–900 nm. The amounts of bacteriochlorophyll and carotenoids were measured as previously described [38].

### 4.3. Determination of RNA Half-Life

To analyze the half-life of specific RNA transcripts, microaerobic precultures of *R. sphaeroides* were diluted to an OD_660_ 0.2 and incubated under phototrophic growth conditions for 8 h (see bacterial strains and growth conditions). After taking sample t_0_ (10–15 mL), rifampicin was added to a final concentration of 0.2 mg mL^−1^. The 10–15 mL samples were taken at indicated time points after addition of rifampicin and harvested by centrifugation (10,000 rpm, 10 min, 4 °C). Afterwards the total RNA was isolated and used for northern blot analysis or real-time RT-PCR.

### 4.4. RNA Isolation

To analyze specific RNA transcripts, microaerobic precultures of *R. sphaeroides* were diluted to an OD_660_ 0.2 and incubated under phototrophic growth conditions for 8 h (see bacterial strains and growth conditions). The cells were harvested by centrifugation at 10,000 rpm for 10 min at 4 °C. For northern blot analysis, quantitative real-time RT-PCR and RNA sequencing of total RNA by the hot-phenol method [39], were used. The RNA was precipitated with 1/10x vol. 3 M sodium acetate pH 4.5 and 2.5x vol. 96% ethanol overnight at −20 °C. For quantitative real-time RT-PCR, the remaining DNA was removed by TURBO DNase treatment (Invitrogen/Thermo Fischer Scientific, Rockford, IL, USA). The absence of DNA contamination was tested by PCR using oligonucleotides targeting *gloB* (RSP_0799) [40]. RNA integrity was tested on a 10% polyacrylamide gel containing 7 M urea and subsequent staining with ethidium bromide, as well as on the Bioanalyzer (described in the data generation description of our deposited RNA-Seq data in the GEO repository). The sequencing libraries were constructed as described earlier [24], using the NEBNext Multiplex Small RNA Library Prep Set for Illumina (NEB, Frankfurt am Main, Germany).

### 4.5. Northern Blot Analysis

For the detection of small RNAs, 8 µg total RNA were separated on a 10% polyacrylamide gel containing 7 M urea and transferred to a nylon membrane (Roth, Karlsruhe, Germany) by semi-dry electroblotting [41]. For detection of the sRNAs, specific oligodeoxynucleotides (listed in Table S2) were labeled with [γ-^32^P]-ATP (Hartmann Analytic, Braunschweig, Germany) by a T4 polynucleotide kinase end-labeling reaction (Fermentas/Thermo Fisher Scientific, Rockford, IL, USA).

For the detection of mRNA transcripts, 10 µg total RNA were separated on a 1% agarose (*w/v*) formaldehyde gel and transferred to a nylon membrane (Roth, Karlsruhe, Germany) by vacuum blotting [12]. For detection of mRNA transcripts, specific PCR products (primer listed in Table S2) were labelled with [α-^32^P]-CTP (Hartmann Analytics, Braunschweig, Germany) by using the Prime-a-Gene Labeling System (Promega, Mannheim, Germany). 

The membranes were hybridized overnight using the Church and Gilbert buffer system [42], washed with 0.01% SDS and 5x SSC in ddH_2_O and exposed on phosphorimaging screens (Bio-Rad, Feldkirchen, Germany). To analyze the intensities of the phosphorimaging signals the 1D-Quantity One software version 4.6.8 Basic (Bio-Rad, Feldkirchen, Germany) was used.

### 4.6. Co-Immunoprecipitation

For CoIP, microaerobic precultures of *R. sphaeroides* WT pRK*ccaF1*FLAG_NT and *R. sphaeroides* pRK*ccaF1* as an untagged control [24] were diluted to an OD_660_ 0.2 and incubated under phototrophic growth conditions for 8 h (see bacterial strains and growth conditions). The cells (from 100 mL culture) were harvested by centrifugation at 10,000 rpm at 4 °C. The CoIP was performed as described in Grützner et al. and Pfeiffer et al. [24,43]. The precipitated CoIP RNA was treated by TURBO DNase (Invitrogen/Thermo Fischer Scientific, Rockford, IL, USA) to remove any DNA contamination. The isolated RNA was analyzed by RNA sequencing and quantitative real-time RT-PCR. 

### 4.7. Quantitative Real-Time RT-PCR

For quantitative real-time RT-PCR, the Brilliant III Ultra-Fast SYBR^®^ Green qPCR Master Mix Kit (Agilent, Santa Clara, CA, USA) was used as described in the manufacturer’s manual. The 10 µL reaction mixtures contained 5 µL Master Mix (supplied), 0.1 µL DTT (100 mM, supplied), 0.5 µL RiboBlock solution (supplied), 0.4 µL water, 1 µL of each primer (10 pmol/L), and 2 µL DNA-free RNA (20 ng/µL). The reactions were performed in a spectrofluorometric thermal cycler (Bio-Rad, Feldkirchen, Germany), and the resulting data were visualized using the Bio-Rad CFX Manager 3.0 software. All real-time RT-PCR experiments were performed in technical duplicates with samples originating from biological triplicates. For all primers, a no-template control was included, to confirm the absence of DNA contamination. RNA abundances were normalized to *rpoZ*. Fold changes were calculated according to Pfaffl [44].

### 4.8. RNA-Seq Data Processing

The RNA-Seq data processing was performed as described in Börner et al. [23]. The alignment of the raw sequencing reads against the reference genome of *R. sphaeroides* (NC_007493.2, NC_007494.2, NC_009007.1, NC_007488.2, NC_007489.1, NC_007490.2, and NC_009008.1) was performed using the READemption pipeline v.1.0.5 [45]. Processed wiggle files were visualized using the Integrated Genome Browser software version 9.1.10 [46].

## Data Availability

The RNA-Seq data are available in the NCBI gene expression omnibus (GEO) repository. The RIP-Seq analysis (CoIP) data are listed in GSE145045 [24] and in GSE230031. The RNA sequencing of the total RNA is listed in GSE200990 [23].

## Supplementary Materials

The supporting information can be downloaded at: https://www.mdpi.com/article/10.3390/ijms24119515/s1. References [12,17,23,24,25,41,47,48,49] are cited in the supplementary materials Tables S1 and S2.
