# Supplementary material for: The Small RNA-Binding Protein CcaF1 Promotes Formation of Photosynthetic Complexes in Rhodobacter sphaeroides
Source: Int J Mol Sci. 2023 May 30;24(11):9515. doi: 10.3390/ijms24119515 (PMC10253847; doi:10.3390/ijms24119515)
Supplement: Supplementary file 1 [file ijms-24-09515-s001.zip › ijms-2421478-supplementary.pdf]

Supplementary Information

Table S1. Bacterial strains and plasmids.

Strain or plasmid	Description	Reference
***E. coli* strains**		
JM109	Cloning strain	New England Biolabs
S17-1	Donor strain for diparental conjugation	(1)
***R. sphaeroides* strains**		
2.4.1	Wild type	(2)
**Plasmids**		
pJET1.2/blunt cloning vector	Ap^r, 2.97 kb	Fermentas
pRK4352	Empty vector control; Tc^r	(3)
pRK*ccaF1*	Constitutive overexpression of CcaF1 (RSP_6037); Tc^r	(4)
pRK*ccaF1*_3xFLAGnt	Overexpression of CcaF1 (RSP_6037) with an N-terminal 3xFLAG-Tag; Tc^r	(4)

Table S2. Oligodeoxynucleotide sequences.

Name	Sequence 5′-3′	Reference
**RT-PCR**		
CcaF1_f	GTCTGGGAGACCGTGTTTCG	This study
CcaF1_r	CCGTAGGCGGCTTCCATC	This study
pufBA_f	ATCACCGCGAGGAGGACAG	(5)
pufBA_r	ACATCTGGCGTCCGTGGTTC	(5)
pufL_f	ACACCTACGGCAACTTCC	This study
pufL_r	ATCGAGTAGCCGACCAGA	This study
pucBA_f	CGAAGCCGAACAAGTTCA	(4)
pucBA_r	TTCACCACGAGCCAGATT	(4)
bchE_f	CGCGATCAGGTCGAGGTCTTC	This study
bchE_r	CCAAGTCGTAGAAGGTTCGCCC	This study
bchB_f	GCTGCTCTACCCCGAGACC	This study
bchB_r	CGAACAGGAACACGCGCTTG	This study
bchN_f	TCGAGGAGAAGGATCTCG	This study
bchN_r	GAGCCGGAGAAGTTGTAGAC	This study
bchL_f	CGATCAGGCGGTGGTGGT	This study
bchL_r	CGTCGAGGTCCGGCATGT	This study
bchM_f	GAGGGTCGCGACACGATGC	This study
bchM_r	GAAGGTCACGCGGTCCTGATG	This study
appA_f	ACGCTTGTTTCAGGGGCT	This study
appA_r	CGCGCAGCGTCAGATCC	This study
fnrL_f	CCGTGGCCTATGACGTGACC	(6)
fnrL_r	GCAGGCTCGCGATCTTCTCG	(6)
ppaA_f	TTTGACGCCATAATGATTTC	This study
ppaA_r	GTCGTTCGTCACGATGTC	This study
ppsR_f	GAGCCGGAGAAGTTGTAGAC	This study
ppsR_r	CATAAAGGGCGAAGGTGT	This study
rpoZ_f	ATCGCGGAAGAGACCCAGAG	(5)
rpoZ_r	GAGCAGCGCCATCTGATCCT	(5)
PcrZ_f	GGAGTGGTAACGAGTATCCGGC	(4)
PcrZ_r	GACCACGCGTATCGAGTCGA	(4)
bchH_f	ATCCCTCCATCGTGAAGCTCAC	(4)
bchH_r	TAGCGGACCATCTGCTCGAGGT	(4)

**Northern Blot**		
p-CcsR1	CGTCGCCGCTGCTGCTACAGGTC	(7)
p-pufBA_f	ACATCTGGCGTCCGTGGTTC	This study
p-pufBA_r	ATCACCGCGAGGAGGAACAG	This study
p-pucBA_f	GCCGAAGAAGTTCATAAGCAA	This study
p-pucBA_r	CAGCCGAGCCTTGGTAGTAG	This study
p5S	CTTGAGACGCAGTACCATTG	(8)
p14S	CTTAGATGTTTCAGTTCCC	(9)

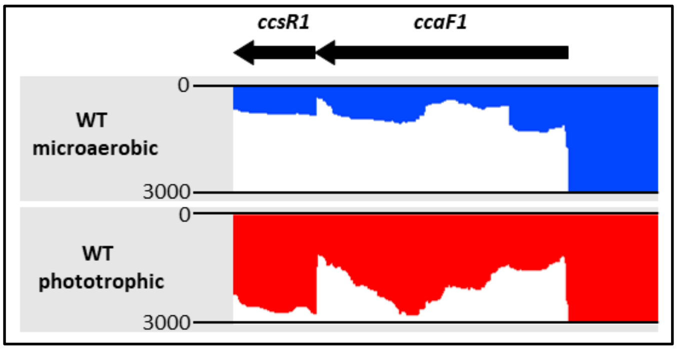

Figure S1. Read coverage of the *ccaF1* mRNA in the *R. sphaeroides* wild type under microaerobic and phototrophic conditions. The screenshot was taken from the Integrated Genome Browser and displays the total read coverage from RNA-seq data obtained from microaerobically (blue) and phototrophically (red) grown wild type of *R. sphaeroides* in exponential growth phase. The merged read coverage of biological triplicates is shown. The read count scale is given as numbers (0-3000).

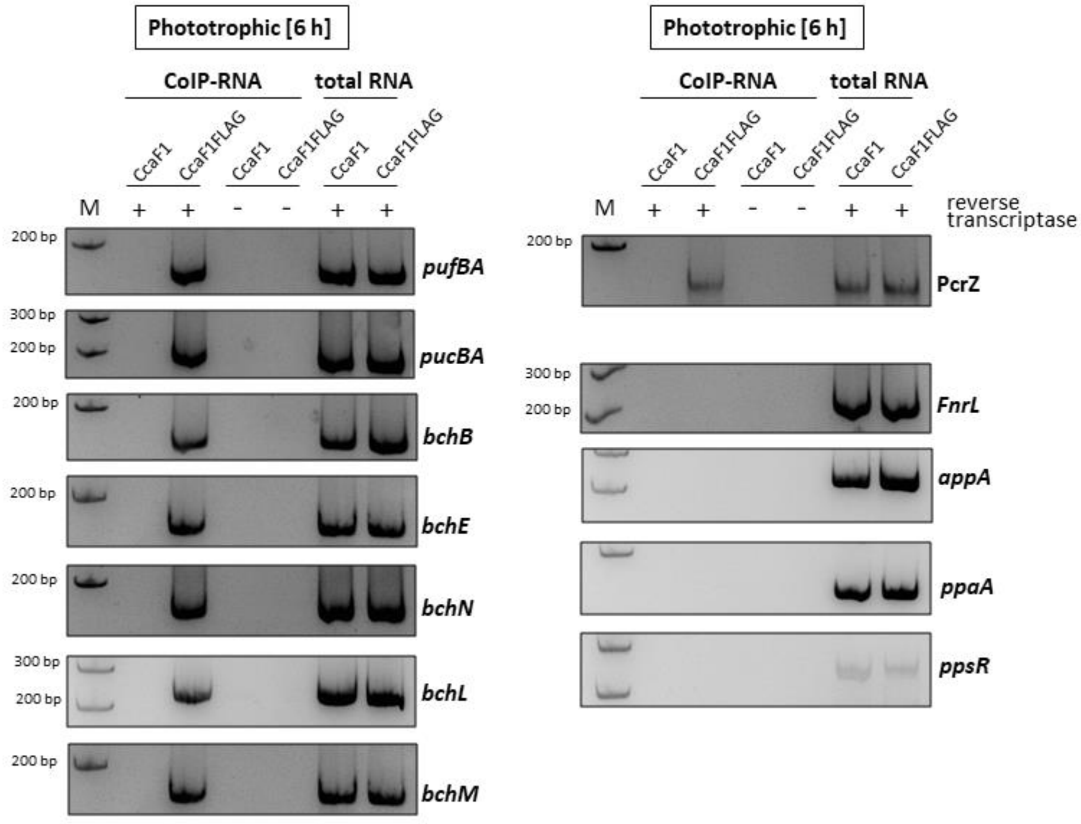

Figure S2. Validation of co-immunoprecipitated RNAs by real-time RT-PCR. The specific non-coding RNAs and mRNA transcripts of the coimmunoprecipitation were analyzed by real-time RT-PCR. The RT-PCR amplicons were separated on a 10% polyacrylamide gel and visualized by ethidium-bromide staining.

References

1. Simon, R.; O´Connell, M.; Labes, M.; Puhler, A. Plasmid vectors for the genetic analysis and manipulation of rhizobia and other gram-negative bacteria. *Meth. Enzymol.* **1986,** *118*, 640-659, doi:10.1016/0076-6879(86)18106-7.
2. van Niel, C.B. The culture, general physiology, morphology, and classification of the non-sulfur purple and brown bacteria. *Bacteriol. Rev*. **1944**, *8*, 1-118, doi:10.1128/br.8.1.1-118.1944.
3. Mank, N.N.; Berghoff, B.A.; Hermanns, Y.N.; Klug, G. Regulation of bacterial photosynthesis genes by the small noncoding RNA PcrZ. *Proc. Natl. Acad. Sci. U. S. A.* **2012**, *109*, 16306–16311, doi:10.1073/pnas.1207067109.
4. Grützner, J.; Billenkamp, F.; Spanka, D.T.; Rick, T.; Monzon, V.; Förstner, K.U.; Klug, G. The small DUF1127 protein CcaF1 from *Rhodobacter sphaeroides* is an RNA-binding protein involved in sRNA maturation and RNA turnover. *Nucleic Acids Res.* **2021**, *49*, 3003–3019, doi:10.1093/nar/gkab146.
5. Jäger, A.; Braatsch, S.; Haberzettl, K.; Metz, S.; Osterloh, L.; Han, Y.; Klug, G. The AppA and PpsR proteins from *Rhodobacter sphaeroides* can establish a redox-dependent signal chain but fail to transmit blue-light signals in other bacteria. *J. Bacteriol.* **2007,** *189*, 2274-2282, doi:10.1128/JB.01699-06.
6. Börner, J.; Friedrich, T.; Bartkuhn, M.; Klug, G. Ribonuclease E strongly impacts bacterial adaptation to different growth conditions. *RNA Biol.* **2023**, *20*, 120–135, doi:10.1080/15476286.2023.2195733.
7. Billenkamp, F.; Peng, T.; Berghoff, B.A.; Klug, G. A cluster of four homologous small RNAs modulates C1 metabolism and the pyruvate dehydrogenase complex in *Rhodobacter sphaeroides* under various stress conditions. *J. Bacteriol.* **2015**, *197*, 1839–1852, doi:10.1128/JB.02475-14.
8. Berghoff, B.A.; Glaeser, J.; Sharma, C.M.; Vogel, J.; Klug, G. Photooxidative stress-induced and abundant small RNAs in *Rhodobacter sphaeroides*. *Mol. Microbiol.* **2009**, *74*, 1497–1512, doi:10.1111/j.1365-2958.2009.06949.x.
9. Braatsch, S.; Gomelsky, M.; Kuphal, S.; Klug, G. A single flavoprotein, AppA, integrates both redox and light signals in *Rhodobacter sphaeroides*. *Mol. Microbiol.* **2002**, *45*, 827–836, doi:10.1046/j.1365-2958.2002.03058.x.
